# Effectiveness of electrical stimulation on nerve regeneration after crush injury: Comparison between invasive and non-invasive stimulation

**DOI:** 10.1371/journal.pone.0233531

**Published:** 2020-05-26

**Authors:** Chanyang Ju, Eunkyoung Park, Taewoo Kim, Taekyung Kim, Minhee Kang, Kyu-Sung Lee, Sung-Min Park

**Affiliations:** 1 Department of Creative IT Engineering, Pohang University of Science and Technology, Pohang, Republic of Korea; 2 Biomedical Engineering Research Center, Smart Healthcare Research Institute, Samsung Medical Center, Sungkyunkwan University School of Medicine, Seoul, Republic of Korea; 3 Department of Medical Device Management and Research, SAIHST, Sungkyunkwan University, Seoul, Republic of Korea; 4 Department of Urology, Samsung Medical Center, Sungkyunkwan University School of Medicine, Seoul, Korea; Wake Forest University School of Medicine, UNITED STATES

## Abstract

Several studies have investigated the use of invasive and non-invasive stimulation methods to enhance nerve regeneration, and varying degrees of effectiveness have been reported. However, due to the use of different parameters in these studies, a fair comparison between the effectiveness of invasive and non-invasive stimulation methods is not possible. The present study compared the effectiveness of invasive and non-invasive stimulation using similar parameters. Eighteen Sprague Dawley rats were classified into three groups: the iES group stimulated with fully implantable device, the tES group stimulated with transcutaneous electrical nerve stimulation (TENS), and the injury group (no stimulation). The iES and tES groups received stimulation for 6 weeks starting immediately after the injury. Motor function was evaluated using the sciatic functional index (SFI) every week. The SFI values increased over time in all groups; faster and superior functional recovery was observed in the iES group than in the tES group. Histological evaluation of the nerve sections and gastrocnemius muscle sections were performed every other week. The axon diameter and muscle fiber area in the iES group were larger, and the g-ratio in the iES group was closer to 0.6 than those in the tES group. To assess the cause of the difference in efficiency, a 3D rat anatomical model was used to simulate the induced electric fields in each group. A significantly higher concentration and intensity around the sciatic nerve was observed in the iES group than in the tES group. Vector field distribution showed that the field was orthogonal to the sciatic nerve spread in the tES group, whereas it was parallel in the iES group; this suggested that the tES group was less effective in nerve stimulation. The results indicated that even though rats in the TENS group showed better recovery than those in the injury group, it cannot replace direct stimulation yet because rats stimulated with the invasive method showed faster recovery and superior outcomes. This was likely attributable to the greater concentration and parallel distribution of electric field with respect to target nerve.

## Introduction

Peripheral nerve injury typically occurs during road traffic accidents, industrial accidents, or injuries sustained at home. Despite recent advances in medical science, complete recovery after peripheral nerve injury is rare and injuries usually result in permanent partial loss of motor function, sensory function, or both [1]. Gordon et al. described three main factors responsible for inadequate functional recovery: (1) slow growth of regenerating axons across the surgical coaptation sites and nerve gaps; (2) decline in the regenerative capacity of the axotomized neurons over time; and (3) the limited time window in which denervated Schwann cells can successfully contribute a regenerative milieu for regenerating axons [2–4]. Post-injury motor and sensory deficit due to inadequate recovery can affect the quality of life. Therefore, development of methods to enhance axonal regeneration is a key imperative to facilitate complete recovery after peripheral nerve injuries.

Several studies have shown that direct electrical stimulation of the injured nerves can enhance sensory and motor axon regeneration, hasten functional recovery, and facilitate reinnervation [2, 5–11]. In a study by Mendonca et al., stimulation of injured peripheral nerves with continuous 1 μA electric current improved functional recovery significantly compared to control groups [5]. Alrashdan et al. observed that 30 minutes of low-intensity electrical stimulation promoted nerve regeneration after crush injury [6]. Furthermore, Koo et al. demonstrated that animals that received electrical stimulation for six days (1h per day) showed superior motor function as compared to those that received stimulation for shorter periods [7].

Demonstration of the beneficial effects of direct electrical stimulation on nerve regeneration after crush injury evoked a considerable interest in developing non-invasive modalities for nerve stimulation with an eye to improve convenience and safety aspects. Various methods have been investigated for treatment of nerve injury, such as muscle stimulation using rubber patch electrodes [12] and nerve stimulation at the site of injury using low-frequency magnetic fields [13] or transcutaneous electrical nerve stimulation (TENS) [14]. These studies showed varying degrees of effectiveness in hastening recovery after injuries; however, most of the studies used different stimulation parameters, such as frequency, amplitude, pulse width, stimulating duration and period, as well as the electrode types. The differences in stimulation parameters used in the studies make it difficult to compare the effectiveness of different parameters as well as the electrode locations. Therefore, an optimal set of therapeutic parameters is yet to be determined [15–17].

There is no clear consensus on the therapeutic effect of different stimulation methods on the recovery of injured nerve. No prior studies have analyzed the effect of different stimulation methods on the regeneration of injured nerves. If invasive stimulation method is better than non-invasive stimulation method (such as TENS) with respect to nerve regeneration, evaluation of its superior effect on the regeneration of injured nerves is of much clinical relevance. In this context, the primary objective of this study was to directly compare the effectiveness of invasive and non-invasive stimulation on nerve regeneration and functional recovery after crush injury.

## Materials and methods

All experimental procedures were reviewed and approved by the Institutional Animal Care and Use Committee of the Samsung Medical Center, Seoul, Korea. All experiments were performed in accordance with the National Institutes of Health Guide for the Care and Use of Laboratory Animals. The animals were housed in individual cages with *ad libitum* access to food and water and exposed to a 12-h light/dark cycle. Eighteen male Sprague Dawley rats (180–200 g) were used in this study. The animals were randomly divided into three groups (*n* = 6 per each group): (1) injury (crush injury, no electrical stimulation); (2) iES (crush injury, invasive and direct electrical stimulation); and (3) tES (crush injury, non-invasive and TENS).

### Surgical procedures

All rats were anesthetized with isoflurane (1 L/min oxygen, 5% isoflurane) initially and with isoflurane (1 L/min oxygen, 1–3% isoflurane) during surgery. The skin was shaved and cleansed with water and alcohol before surgery. The shaved area was larger than the patch electrode used in the TENS stimulation group. A 2-cm long incision was made to expose the sciatic nerve of the left hind leg. Two kinds of operative procedures were performed: an injury operation (crush injury, *n* = 12) and an implantation operation (crush injury with implantable stimulator, *n* = 6). Double suturing using an interrupted semi-mattress suture and an American suture was performed on the incision site to prevent wound reopening. Furthermore, the incision site was disinfected daily to prevent possible damage. The injury operated animals were divided into the injury group and the tES group. Crush lesions were inflicted twice on each rat’s left sciatic nerve with a needle holder for 3 s each. A customized cylindrical nerve cuff electrode (inner diameter 2 mm, outer diameter 3 mm, length 5.25 mm, Microprobes Inc., MD, USA) and a miniatured coil to receive wirelessly transmitted power were completely implanted afterwards, covering both crush lesions. The right hind leg was not operated and its gastrocnemius muscle and sciatic nerve served as within-animal controls. Antibiotic prophylaxis (Baytril, 5 mg/kg) was administered postoperatively to prevent wound infection.

### Electrical stimulation procedures

**Fig 1:**
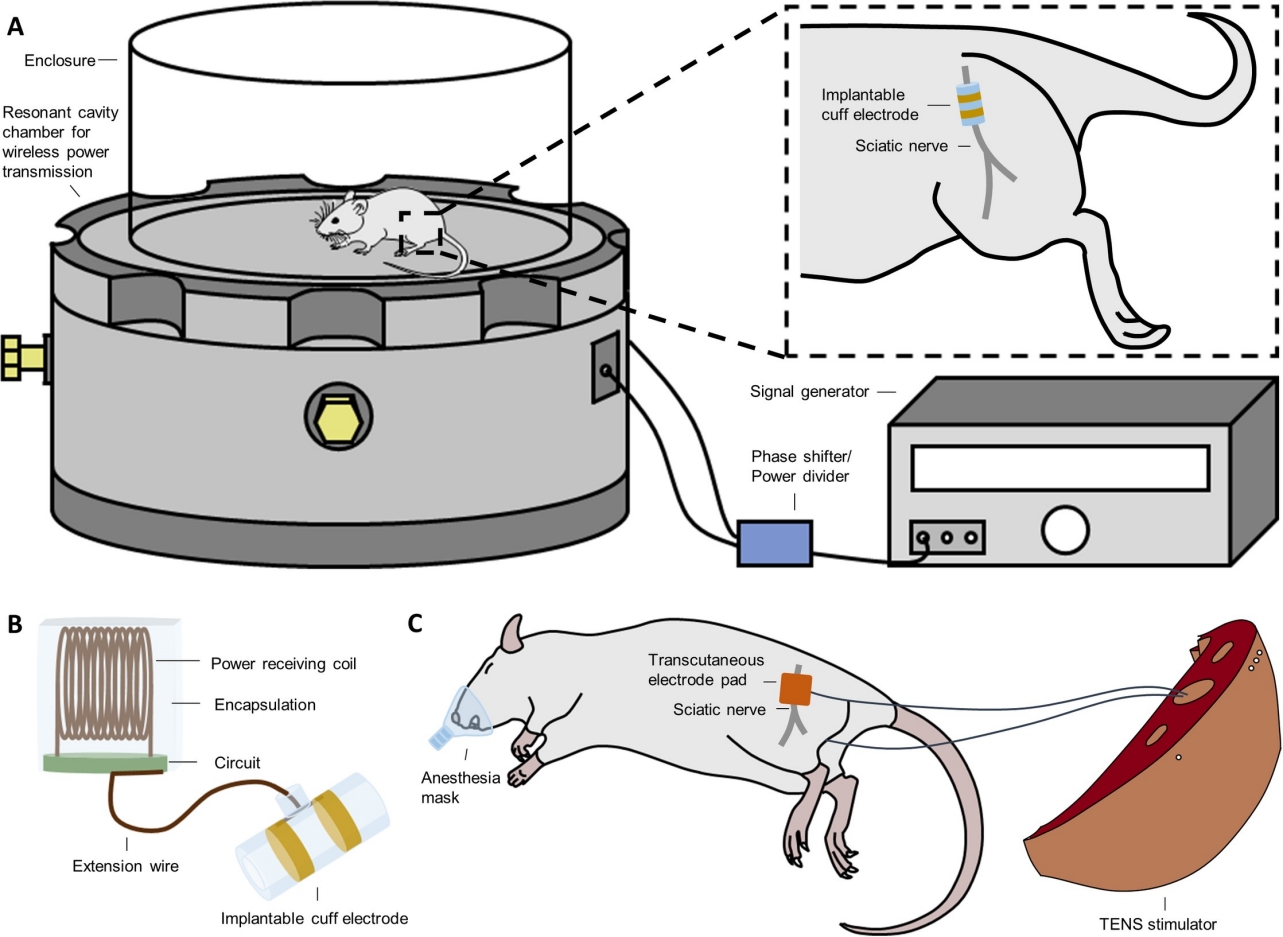
Schematic illustration of electrical stimulation system. A) Wirelessly powered stimulation system for nerve stimulation in the iES group; magnified image showing the implanted cuff electrode (inner diameter 2 mm, outer diameter 3 mm, length 5.25 mm) situated on the sciatic nerve. B) Schematic of the completely implantable stimulation system. C) 7 × 7 mm copper electrode attached just above the femur bone and inner thigh and connected to NuEyne TPD-NH1 for stimulation in the tES group.

Rats in the iES group received invasive electrical stimulation treatment using fully implantable and wireless power transmitted stimulators and cuff electrodes. The fully implantable stimulator used in this study represented a refined version of the device developed by Montgomery [18]. Therapeutic electrical stimulation (25Hz, 2–3 V, pulse width 0.1 ms) was applied to the animals via the implanted stimulator without anesthesia (Fig 1A and 1B). In the tES group, the electrodes were positioned on the animal’s back and inner thigh to generate an electric field between the two electrodes around the injured nerve (Fig 1C). All animals were shaved and cleansed prior to electrode placement and a gel conductor layer was applied between the electrodes and skin for attachment. The sciatic nerve was then stimulated by applying therapeutic electrical stimulation (25 Hz, 1–3 mA, 0.1 ms pulse width), using the customized device from TPD-NH1 (NuEyne, Seoul, Korea). The animals were anesthetized with isoflurane (1 L/min oxygen, 1–3% isoflurane) during tES stimulation. Visible muscle contraction on the hind paw of the animal was set as the threshold for the intensity of electrical stimulation. All animals in the iES and tES groups received 30 min of stimulation every day, five times a week, for 6 weeks from the injury day because recovery of the motor function tends to saturate after 5 weeks [13].

### Functional evaluation

Sciatic functional index (SFI) was used to assess nerve function recovery after inducing crush lesions in the rats. All rats were trained for the test for a week before surgery. The rats were evaluated every week, including on the day of the surgery.

The SFI value was calculated using the equation described by Bain et al. [19]. The animals were coerced to walk through a confined corridor, which was 50 cm long and 15 cm wide, with white paper placed on the floor and sheets covering the top and the end of the walkway. The hind paws of the rats were pressed onto an ink-soaked sponge to retain their footprints on the white paper; the injured left paw print provided experimental data and the right paw print provided normal data. Three parameters were measured from the footprints: (1) the print length (PL); (2) the toe spread (TS); and (3) the intermediary toe spread (IT). All three measurements were obtained from the experimental (E) and normal (N) sides. The SFI was calculated using the following equation:

$$SFI=-38.3\;\left(\frac{EPL-NPL}{NPL}\right)+109.5\;\left(\frac{ETS-NTS}{NTS}\right)+13.3\;\left(\frac{EIT-NIT}{NIT}\right)-8.8$$ (1)

Every 2 weeks after surgery, two rats from each group were euthanized with CO_2_ and the sciatic nerve and the gastrocnemius muscle were dissected from both legs. The nerve and muscle from the right leg served as within-animal controls. The samples were immediately immersed in a 4% paraformaldehyde solution and stored at 4°C for fixation.

### Histomorphometric analysis

The gastrocnemius muscle samples were dehydrated, transversely cut into 1-μm thick sections, and stained with hematoxylin and eosin (H&E). The sciatic nerve samples were dehydrated, penetrated with propylene oxide, and polymerized. They were cut into 1-μm thick sections using a microtome (Leica EM UC6, Leica Biosystems, Mount Waverley, Australia) with a diamond knife and stained with toluidine blue. Photos of the gastrocnemius muscle at ×200 magnification were obtained using an optical microscope (Olympus IX70; Olympus Optical Co, Ltd., Tokyo, Japan). The area of the muscle fiber was calculated in three different randomly selected areas using ImageJ software (National Institutes of Health, Bethesda, MD, USA). Likewise, photos of the sciatic nerve at ×400 magnification were obtained to measure the axon diameter and g-ratio from three different random areas of 90,000 μm^2^, accounting for at least 30% of the total area. From each randomly selected area, the axon diameter (α) and the shortest diameter of the myelinated nerve fiber (β) were measured. The g-ratio, which is the ratio between the inner and outer diameter of the myelin sheath, was calculated using the following formula: α/β.

### Electric and vector field simulation

A biomedical electromagnetic simulator (Sim4Life, Zürich MedTech AG, Switzerland), which is a virtual simulation tool to simulate electromagnetic pulse penetration on an anatomical model, was used to simulate the electric field and vector field distribution in the 3D rat anatomical model, a *big male rat* (Sprague Dawley, 567 g) [20]. The 3D anatomical model, which is a computational animal phantom with realistic geometry and tissue properties, was virtually generated based on MRI images taken from the Sprague Dawley rat. The respective tissue parameters were set at a frequency of 25 Hz with the parameters shown in Table 1 [21]. Simulation was conducted under electro-quasi-static assumption.

**Table 1:** Material properties for simulation at 25 Hz.

Material	25 Hz	
	Conductivity σ (S/m)	Relative Permittivity *ε_r_^a^*
Air^b^	0	1
Skin	2 × 10^−4^	1135.98
Fat	1.6686 × 10^−2^	3.9622 × 10^6^
Muscle	0.211123	2.33605 × 10^7^
Blood	0.7	5259.95
Gel	0.6	76.5

^a^Relative permittivity *ε_r_ = ε/ε*_0_

^b^Background

E=−∇ϕ. (2)

**Fig 2:**
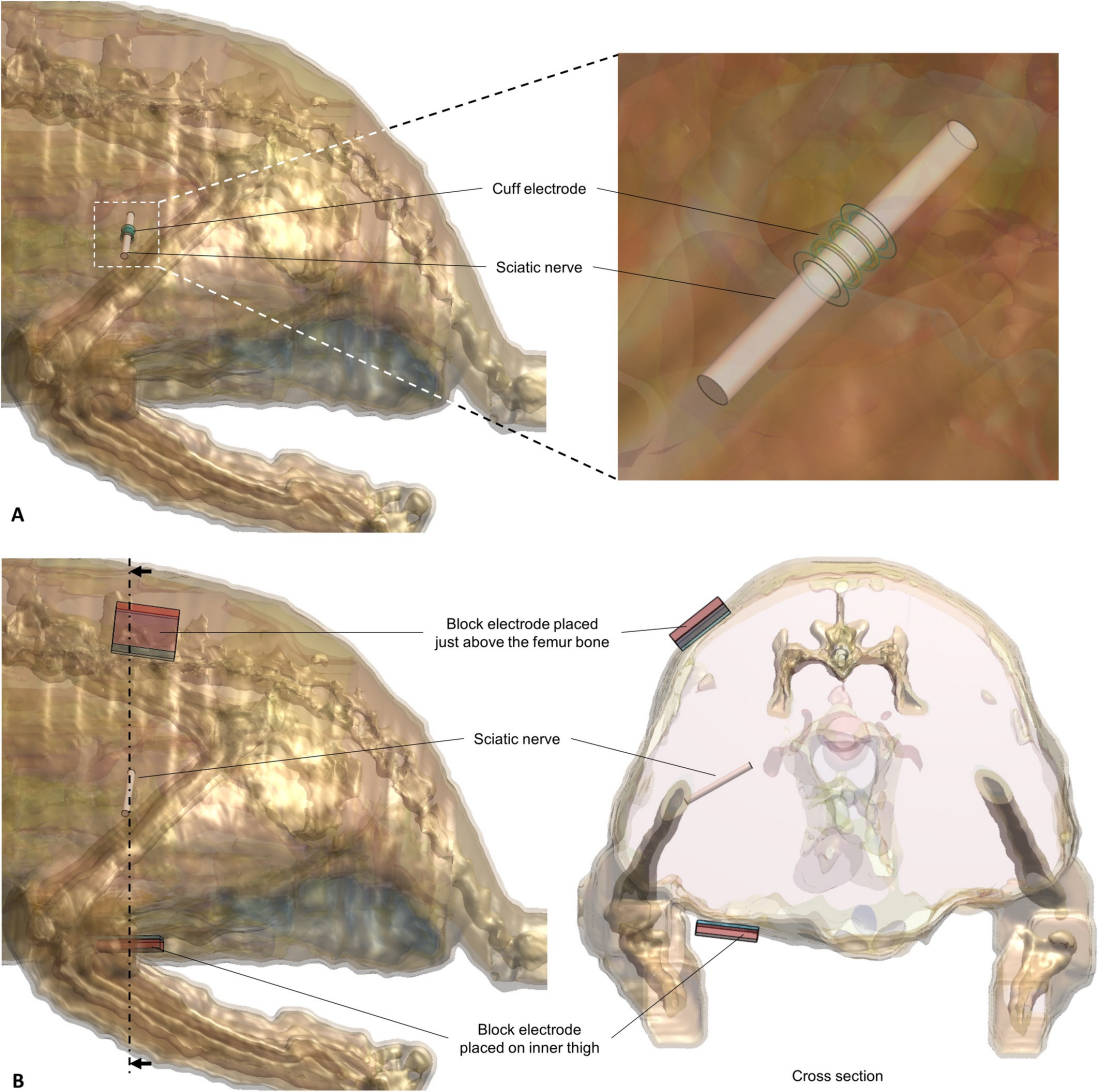
Electrode placed on 3D rat anatomical model, a *big male rat*, simulating experimental setup. A) iES group simulation model; an enlarged image showing the cuff electrode placed on the sciatic nerve. B) tES group simulation model; block electrodes placed just above the femur bone and inner thigh (left) and a cross section showing the block electrodes attached to the skin and sciatic nerve (right).

Wherein Laplace’s equation was solved using the Dirichlet constant voltage boundary condition without the source current:

$$\nabla ∙[(\sigma +j\omega \varepsilon )\nabla \phi ]=0,(∵J_{source}=0),$$ (3)

where ε is the permittivity, σ is the effective electrical conductivity, and ω is the angular frequency. Fig 2 shows the electrodes positioned on the rat model for both invasive and non-invasive stimulation. For the iES group modeling, a virtual cuff electrode was placed on the most probable sciatic nerve area (Fig 2A). A constant voltage of 2V from a single voltage source was used for the simulation parameter as the average voltage transmission to the cuff electrode in the experimental setting (2V) was measured beforehand. In contrast, for the tES group modeling, two virtual electrodes were placed at the back of the rat and inner thigh (Fig 2B) to imitate electrode placement in the experiment. The load voltage between the electrodes was 17 V peak-to-peak to simulate a 5 mA current source.

After electromagnetic pulse penetration in the model was calculated, the simulated electric fields were extracted along the xy- and yz-planes to determine the field distribution. Electric field intensity applied to the sciatic nerve was calculated by averaging the value obtained from 15 voxels in the center of the chosen cross section. The cross section located equidistantly from the two-metal ring of the cuff electrode was chosen. The vector fields were extracted along the xy-plane, which were parallel to the sciatic nerve spread, to determine the field direction along the nerve growth direction.

### Data analysis

SFI data is presented in scatter plot and fitted to the Boltzmann equation, owing to the varying sample size over time. The SFI values obtained from the fitted equation were used to calculate the span of improvement in functional regeneration, by subtracting minimum (week 1) value from maximum (week 6) value. The non-parametric data were represented as median and quartiles. Owing to the small sample size, histomorphometric data is presented in boxplot with individual data from each group presented in dotted boxplot. The data was also linearly fitted to compare recovery rate between the groups with its slopes.

## Results

### Functional evaluation

**Fig 3:**
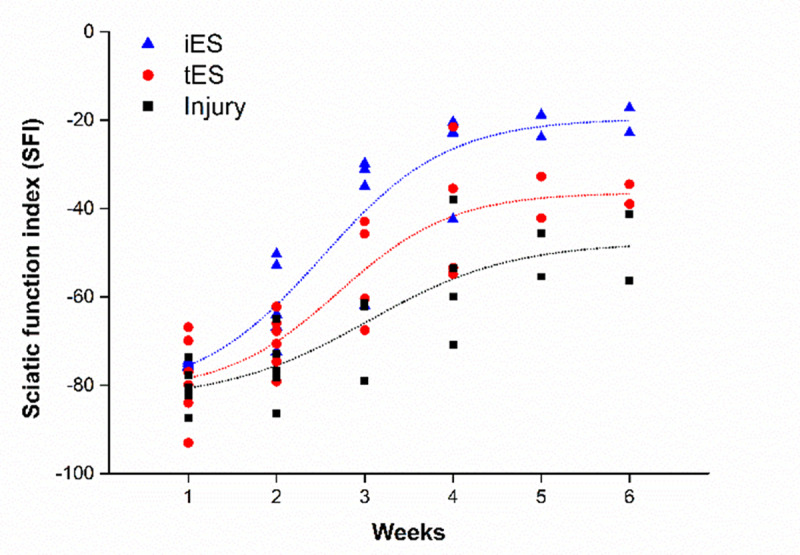
Temporal change in Sciatic Functional Index (SFI) in each group. Functional recovery measured every week starting a week after surgery using the toe-spreading SFI. The dot lines on the graph was best fitted of SFI data points of each group using Boltzmann equation.

Motor function was evaluated using the SFI. The test indicates normal neurological function when the SFI is near 0 and impaired neurological function when the SFI is near -100. The same observer analyzed the hind paw prints obtained weekly, including the preoperative records, during the study. The SFI was represented with Boltzmann equation as non-linear regression sigmoidal fit. The R-squared values of all groups, 0.999, support the feasibility of fitting models. The mean SFI value in all experimental groups showed a sharp decline after the crush injury reaching −80 and then gradually increasing over 6 weeks (Fig 3). However, even though the three groups showed a similar regeneration trend, differences were observed with respect to the degree of recovery. SFI values in each group improved from the first to sixth week, with span of 62.39, 44.92, and 35.59 in the iES, tES, and injury groups, respectively; this reflected distinct differences in the improvement of motor function. Moreover, after 6 weeks of the experiment, the mean SFI values in the iES, tES, and injury groups were -19.66, -36.47, and -47.70, respectively, showing that the iES group attained better regeneration than the other groups.

### Histomorphometric evaluation

#### Nerve histology

**Fig 4:**
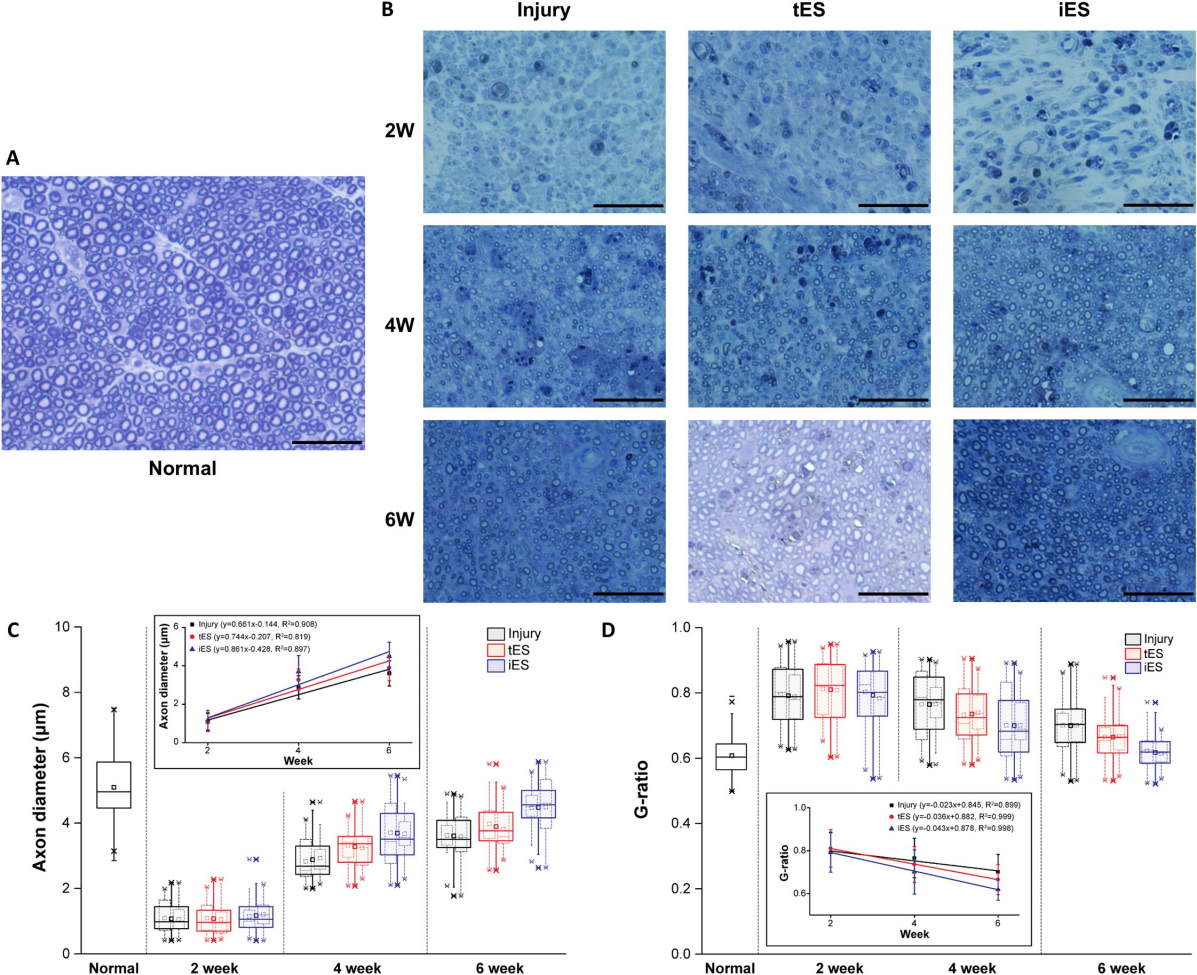
Histomorphometric evaluation of sciatic nerve section. Transverse nerve sections stained with toluidine blue from A) normal and B) each group over time. C), D) Axon diameter and g-ratio of each group over time represented in boxplot (solid line); individual data of each group were indicated as dotted boxplot; the inset graph showed improvement trends of axon diameter and g-ratio over time by linearly fitted lines. The horizontal line and square inside each box indicated the median and mean, respectively; the upper and lower borders of each box represented the first and third quartile; the whiskers indicated 1th and 99th percentiles. Bar = 50 μm.

The histologic findings (Fig 4) supported the tendency observed in the functional evaluation. Compared to the regular distribution of small and large diameter nerve fibers in normal nerves, the cross-sections of the crushed nerves were dominated by small diameter fibers with thin myelin sheath after 2 weeks. Increasing numbers of larger diameter thick myelin sheath fibers were observed at subsequent weeks. Furthermore, the iES group was most similar to the normal group among the injured groups and fibers in the tES group had relatively larger axons and thicker myelin sheaths than the injury group. These observations reflected the same trend of nerve regeneration as observed in the functional evaluation (Fig 4A and 4B).

The axon diameters (Fig 4C) and the g-ratio (Fig 4D) were evaluated in the injury, iES, and tES groups every 2 weeks after surgery. At week 2 after surgery, the axon diameters from every group diminished without significant between group differences. The axon diameters in each group improved over time, with slopes of 0.861, 0.744, and 0.661 in the iES, tES, and injury groups, respectively; this reflected distinct differences in the speed of improvement with iES group recovering faster than the other two groups. The optimal value of the g-ratio, which is the ratio between the inner and the outer diameter of the myelin sheath, is 0.6 for peripheral nerves [22]. Over time, the g-ratio of all groups approached the optimal value with slopes of -0.043, -0.036, and -0.023 in the iES, tES, and injury groups, respectively (Fig 4D); this showed the same trend of iES group recovering faster than the other two groups.

#### Muscle histology

**Fig 5:**
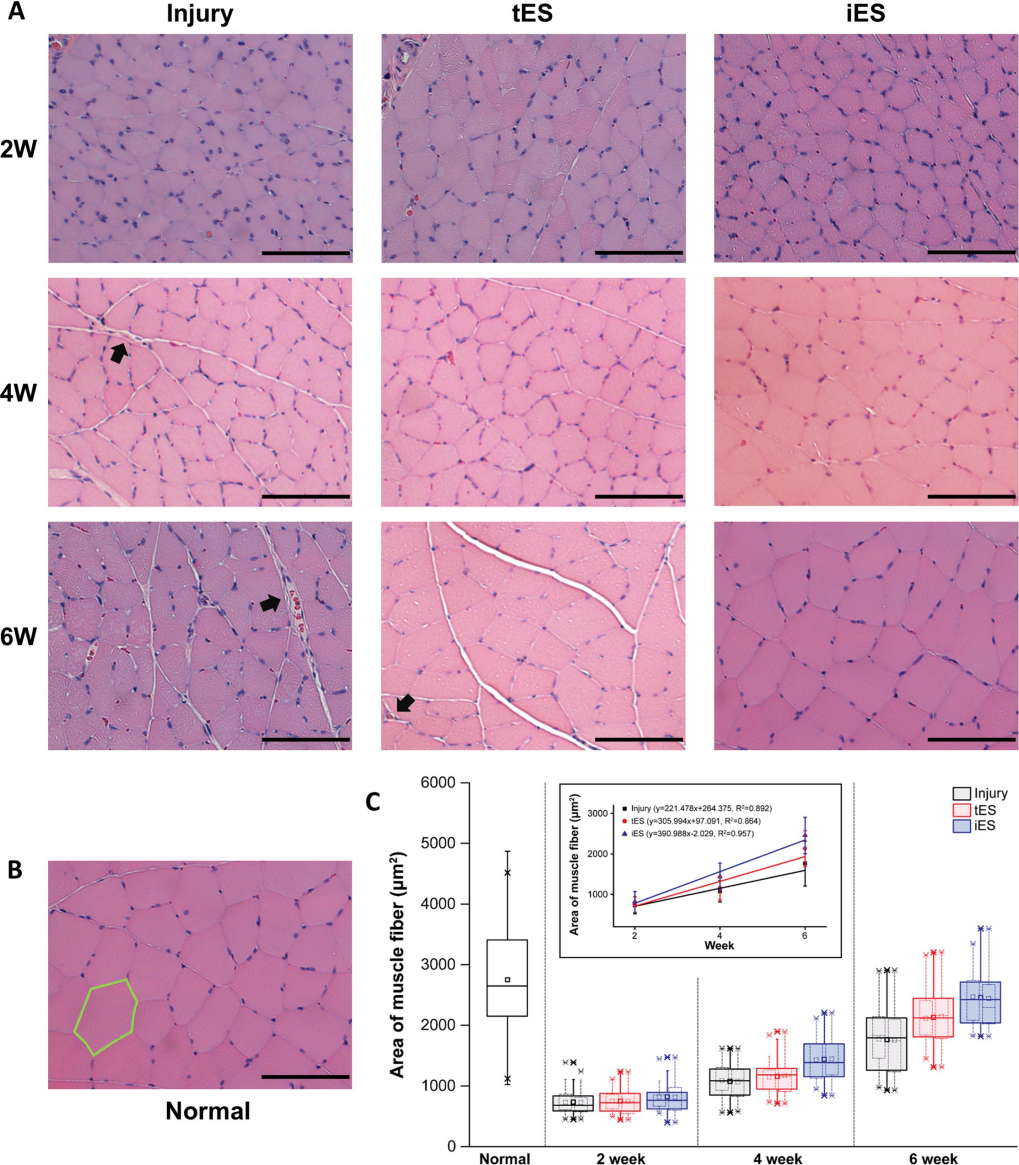
Histomorphometric evaluation of gastrocnemius muscle sections. A) Transverse muscle sections from each group over time; black arrows indicate fibrosis. B) Transverse muscle section from normal rat with H&E staining; green polygon indicates myofiber. C) muscle fiber area of each group over time presented in boxplot; linearly fitted muscle fiber area enclosed in the graph. Bar = 100 μm.

Representative microscopic images of the transversely cut gastrocnemius muscle from each group obtained every two weeks are shown in Fig 5. Compared to normal muscle fibers, the muscle fibers from the injured legs were all smaller and rounder, especially in the earlier weeks. The histological characteristics of muscle fibers in the iES group at week 6 were similar to those of muscle fibers in the normal group. The area of the muscle fibers in the injury and tES groups increased over time, but showed signs of fibrosis, as well, compared to the normal and iES groups (Fig 5A and 5B). Fig 5C shows the same trend. The area of the muscle fibers diminished sharply after surgery and increased over time with slopes of 390.988, 305.994, and 221.478 in the iES, tES, and injury groups, respectively. The difference between slopes reflected same trend of recovery as observed in previous results.

### Electric and vector field simulation

**Fig 6:**
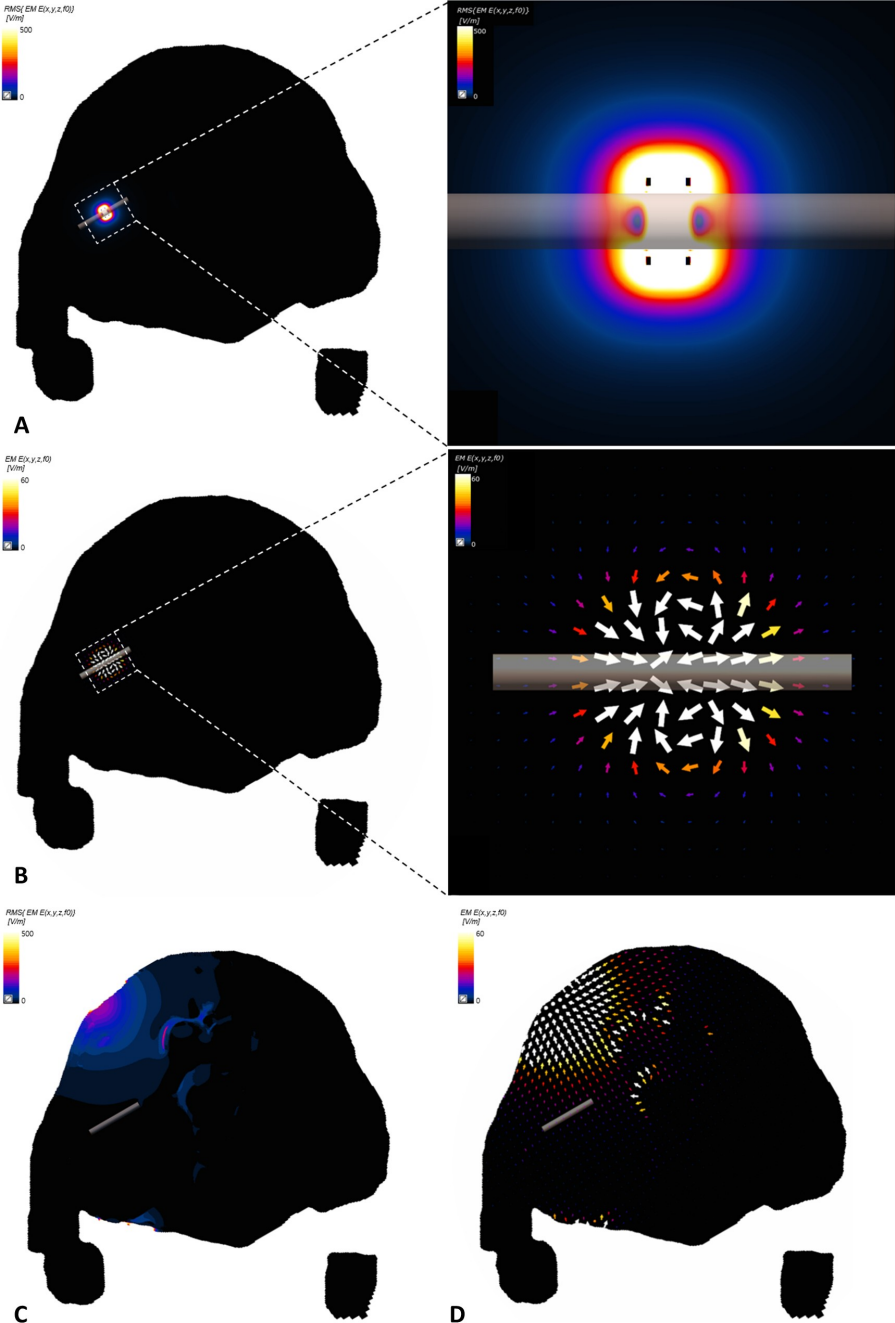
Simulated E-field and vector field distribution. E-field distribution expressed on the cross section of the *big male rat* for A) direct stimulation case and C) TENS case; color map corresponds with the E-field intensity, with black as zero; and vector field distribution for B) direct stimulation case and D) TENS case; arrows indicate the direction of the E-field; the color of the arrow corresponds with E-field intensity. The cross section corresponds with the section shown in Fig 2B, passing through the sciatic nerve of the *big male rat*. Transparent tube represents the sciatic nerve.

The electric and vector field simulations for both the iES and tES groups were performed using the Sim4Life (Zürich MedTech AG) biomedical electromagnetic simulator. Fig 6 shows the cross section of the simulated E-field distribution induced by the stimulation currents inside the rat’s body, as well as the vector field distribution between or around the electrodes, which are visualized by calculating the results from Eqs (2) and (3). With the iES group simulation (Fig 6A), the E-field was highly localized around the cuff electrode, which was positioned on the sciatic nerve. Due to the high concentration around the electrode, the highest E-field intensity was obtained near the two-ring electrodes of the implanted cuff electrode. Mean E-field intensity inflicted on the chosen section of the sciatic nerve was 1039 V/m. In contrast, the E-field of the tES group simulation (Fig 6C) was concentrated on the rat’s skin near the attached electrodes, which generated low E-field intensity (23 V/m) on the chosen section of the sciatic nerve. The E-field intensity on the sciatic nerve was almost fifty times lesser than that in the direct stimulation. Furthermore, the vector field distribution (arrows in Fig 6B) indicates that in the direct stimulation case, the electrical field developed along the sciatic nerve, parallel to its spread; in contrast, in the TENS case, the vector field developed between the two electrodes attached on the skin, showing an orthogonal distribution to the sciatic nerve (Fig 6D).

## Discussion

Various methods and parameters have been proposed for non-invasive stimulation to enhance peripheral nerve regeneration; however, its effectiveness compared with that of invasive stimulation is not well characterized. Therefore, whether non-invasive stimulation can replace conventional invasive methods is not clear. Previous studies that compared invasive vs. non-invasive stimulation employed different parameters, such as frequency, stimulation time, and stimulation period; this prevented any meaningful comparisons between the two modalities. Thus, we compared the effect of direct stimulation on nerve regeneration using fully implantable electrode and TENS under similar parameters in a reproducible and controlled environment and evaluated the temporal changes in function and histological characteristics.

Numerous studies have reported that sciatic nerve regeneration can be accelerated by direct electrical stimulation to the injured site. The stimulated regions showed increased expressions of brain derived neurotrophic factor and tyrosine receptor kinase B which reduced the susceptibility of the growth cones at the injured site, promoting nerve regeneration [9, 23, 24]. Application of electrical field during nerve growth has been shown to directly affect nerve branching [25, 26]. These results provided the scientific rationale behind the positive effect of electrical stimulation on recovery, and are consistent with the results of functional and histomorphometric evaluation in the present study.

In this study, we performed functional evaluation using the SFI method. The reliability and reproducibility of this method for evaluating the hind paw motor function of rats has been demonstrated [27]. Hind paw prints of the two stimulation groups and one control group were measured every week, starting from one week after surgery. Generally, the normal SFI value is approximately 0. The SFI value of rats with nerve injury shows a sharp decline one week after injury followed by a gradual increase from week 2 onwards [5, 28]. Fig 3 shows the same trend; the mean SFI value in the first week among the groups was approximately -80, which implied that the animals were almost unable to stand on their injured paw. This was followed by a progressive increase in SFI over successive weeks. SFI data is fitted to the Boltzmann equation with R-squared value of 0.999 in all groups. The span of improvement is calculated using the SFI values obtained from the fitted equation, by subtracting minimum value (week 1) from maximum (week 6) value. Each group showed an improved sciatic function from the first to sixth week, with span of 62.39, 44.92, and 35.59 in the iES, tES, and injury groups, respectively. The greatest increase in SFI value was observed in the iES group, with the tES group showing greater increase than the injury group. The results indicated that the regeneration efficiency in the iES group was greater than that in the tES group since faster increase in SFI values imply faster and better recovery. Furthermore, the mean SFI values in the iES, tES, and injury groups on week 6 were -19.66, -36.47, and -47.70, respectively, with a sign of saturation around week 5. A similar trend of saturation around week 5 was previously reported with crush injury regeneration [13]. The signs of saturation observed from weeks 5 and 6 can be interpreted as superior regeneration outcomes in the iES group compared to the other two groups. Thus, not only did the iES group show a faster regeneration rate during recovery, but it also exhibited superior functional outcomes at the end of the experiment.

The results of the histomorphometric evaluation also indicated that electrical stimulation of the injured nerve accelerated neural regeneration. The stimulated neurons showed larger axon diameters and g-ratio closer to 0.6, as previously reported [5, 6, 8, 22]. However, a previous study on the histomorphometric features of TENS-treated injured nerves reported different histological and morphological results according to the stimulation frequency; the results suggested that low-frequency stimulation was better than high-frequency stimulation [14]. The nerve sections stimulated by relatively low frequency are shown in Fig 4; while all groups showed a steady recovery as indicated by improved parameters, the iES group had larger axon diameters than the tES group at week 4; the differences became more distinct at week 6. The tES group also tended to have larger axon diameters than the injury group, which proved the beneficial effects of electrical stimulation on regeneration. The differences in slopes with the value of 0.861, 0.744, and 0.661 in the iES, tES, and injury groups, respectively, indicates the same tendency; the iES group has largest slope and the tES group has larger slope than the injury group. Slopes in g-ratio with the value of -0.043, -0.036, and -0.023 in the iES, tES, and injury groups, respectively, showed the same trend, as well, with the g-ratio of the iES group declining faster than the other two groups. Furthermore, although the g-ratio approached 0.6 in all groups over time as a sign of regeneration, only the iES group showed considerable difference compared to the other groups at week 6 and had a similar g-ratio with normal. This not only indicates better recovery of iES groups than the tES or injury group after 6 weeks of stimulation but also shows the possibility of the iES group regaining the nerve similar to that of normal at the end. These results show the tendency of better neural regeneration in the iES group than in the other groups in the morphological aspects.

The iES group had a larger area of muscle fiber than the tES group at weeks 4 and 6, and the tES group had a larger area of muscle fiber than the injury group at week 6. Besides the fact that the iES group lacked signs of fibrosis, which could happen with delayed muscle regeneration [29], and showed similar histological features as the normal group at week 6, histological evaluation of the muscle also showed the tendency of invasive stimulation to be more efficient than TENS for neural regeneration. Furthermore, the area of the muscle fibers increased over time with slopes of 390.988, 305.994, and 221.478 in the iES, tES, and injury groups, respectively; this observation reflected the same trend of recovery with previous results. The rapid increase in the muscle fiber area between weeks 4 and 6 differed from the histological parameters, which increased rapidly between weeks 2 and 4; this implies that muscle fiber regeneration and reinnervation starts after progression of axonal regeneration. The results suggest that faster nerve regeneration helps prevent further muscle atrophy and ensures better and faster muscle fiber regeneration, leading to better functional outcomes.

All experimental results indicated that electrical stimulation facilitates regeneration of injured nerve; direct stimulation caused better recovery than TENS with respect to functional and morphological parameters during the six weeks of the experiment. Furthermore, iES group showed faster recovery in every aspect and better recovery of motor function at the end of the experiment. This indicates that direct stimulation is much more preferable than TENS as it hastens nerve regeneration and facilitates greater functional recovery after injury.

To understand why the iES group showed better efficacy than the tES group, simulations were performed for both groups. In the results section, we detailed the simulated E-field distribution inside the rat body as well as the vector field distribution between and around the electrodes. With direct stimulation using the cuff electrode, the E-field was concentrated around the electrode positioned on the sciatic nerve, which enabled high E-field concentration and intensity around the site of injury with a relatively smaller risk of side effects. In contrast, the E-field was concentrated along the electrodes attached to the rat skin with TENS, which resulted in lower E-field intensity near the sciatic nerve compared to direct stimulation. The higher concentration and intensity of the E-field near the sciatic nerve in the case of direct stimulation might be one of the factors influencing the differences in efficiency, especially since the E-field intensity induced by TENS has an upper limit due to skin irritation at higher settings. Moreover, the vector field distribution in both cases may also have influenced the results. In the case of direct stimulation, the electrical field developed along the sciatic nerve, parallel to its spread, whereas the vector field between the two electrodes attached to the skin in the TENS case was distributed orthogonally to the sciatic nerve. Several studies have reported that the nerve sensitivity in parallel fields is greater than that in orthogonal fields, suggesting that the neurons of the tES group were less susceptible to stimulation than those of the iES group [30, 31]. This result may attribute to the paradigm of nerve stimulation for greater recovery by suggesting the need to focus electric field around the sciatic nerve and to align the vector field with the sciatic nerve spread.

In conclusion, invasive and non-invasive electrical stimulation of injured sciatic nerve can enhance nerve regeneration and hasten functional recovery. However, functional evaluation and histomorphometric analysis showed that rats subjected to direct stimulation by implanted electrodes after crush injury displayed more enhanced regeneration than rats with TENS stimulation. The simulated E-field and vector field distribution of both methods suggest the need for focused E-fields in the targeted area and for calculated electrode placement to facilitate spread of E-field along the sciatic nerve spread. This is easily achievable with implantable electrodes; however, since an additional surgery is required for subsequent removal of the electrodes, development of clinically approved bioresorbable electrodes would be more beneficial [7]. However, to also avert the initial implantation surgery, there is a need to develop novel non-invasive stimulation methods, such as temporal interference stimulation to achieve nerve regeneration [32]. In the meantime, direct stimulation of injured nerves should be used for better recovery. Further in-depth studies with larger group of animals are required to determine the optimal parameters and to develop devices for clinical application.
